# Supplementary material for: Integrated analysis of single-cell embryo data yields a unified transcriptome signature for the human pre-implantation epiblast
Source: Development. 2018 Feb 1;145(3):dev158501. doi: 10.1242/dev.158501 (PMC5818005; doi:10.1242/dev.158501)
Supplement: Supplementary information [file develop-145-158501-s1.pdf]

Supplementary Figures

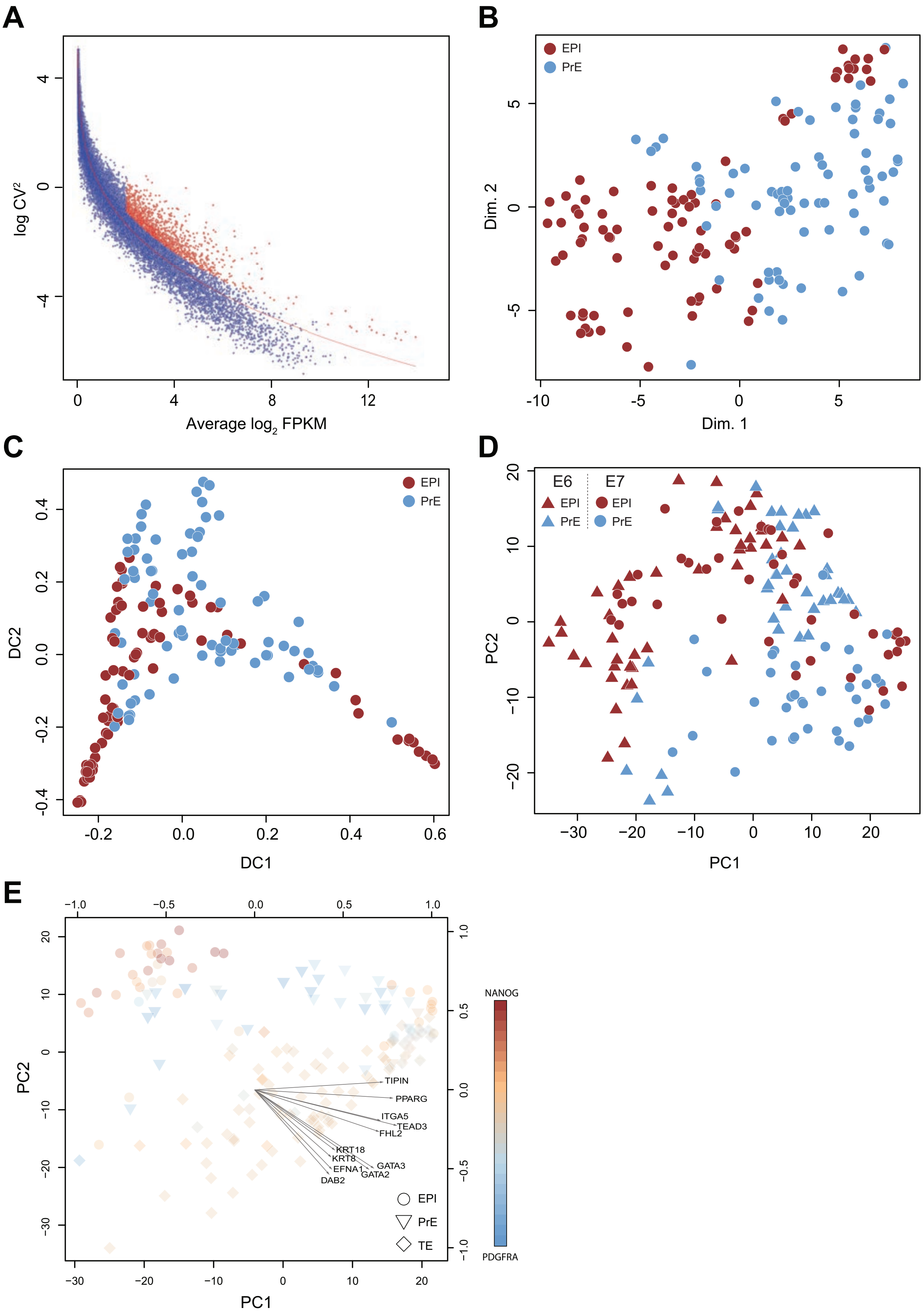

Fig. S1: (A) Highly variable genes identified in E6 and E7 single-cell transcriptome data classified as EPI or PrE in Petropoulos et al. (2016). (B) t-SNE plot and (C) diffusion map of E6 and E7 EPI and PrE samples. (D) PCA of samples identified as EPI or PrE by Petropoulos et al. with developmental stage indicated. (E) Contribution of TE marker expression in Petropoulos immunosurgery samples.

Development • Supplementary information

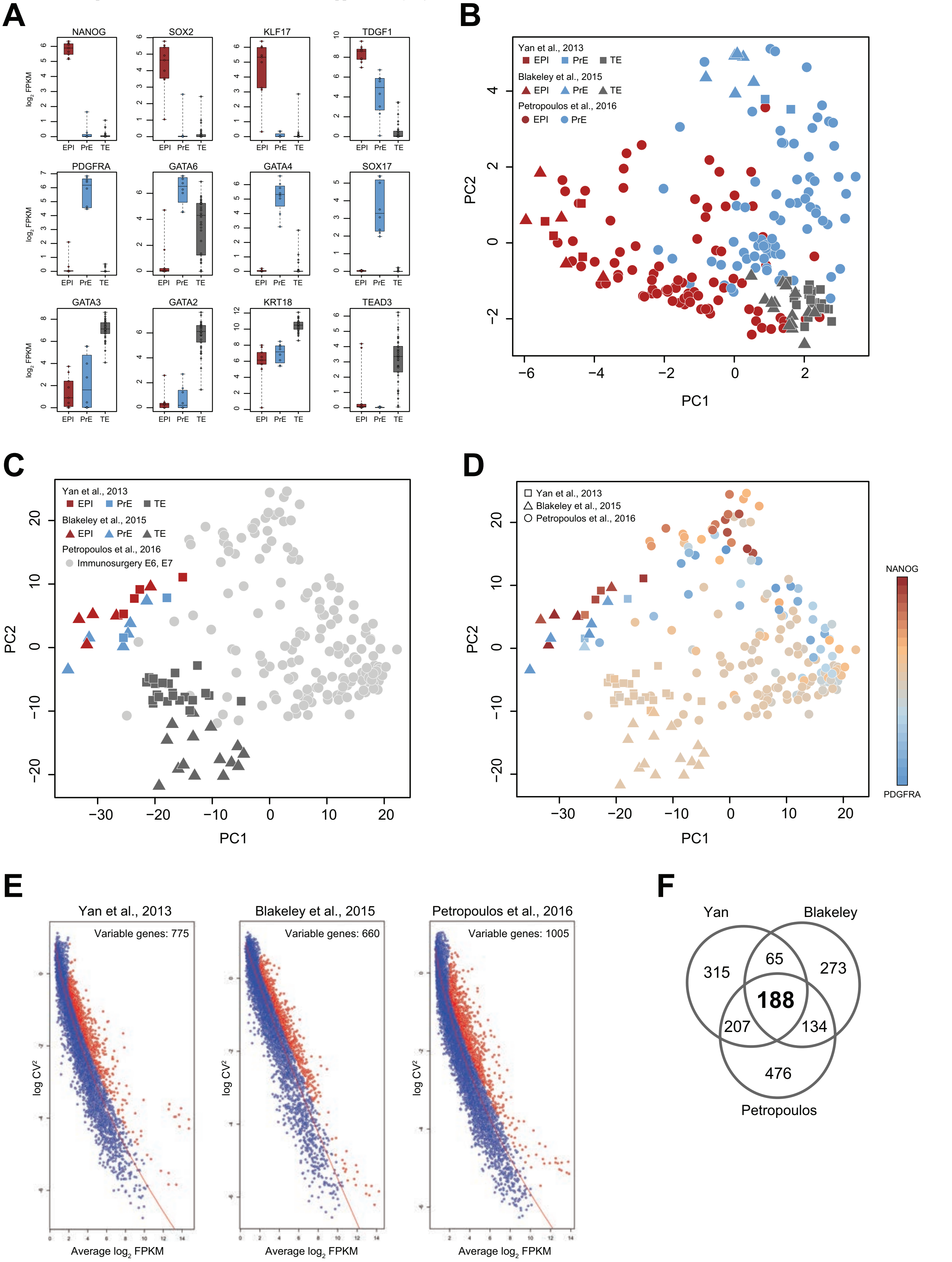

Fig. S2: (A) Expression (log_2 FPKM) of high-confidence lineage markers in cells profiled in Blakeley et al. (2015). (B) PCA of EPI, PrE and TE from Yan et al. (2013) and Blakeley datasets, together with E6 and E7 EPI and PrE samples from the Petropoulos study. (C) PCA based on variable genes (*n*=920) for EPI, PrE and TE samples from Yan and Blakeley datasets with the E6 and E7 immunosurgery subset from Petropoulos et al. (grey). (D) PCA as in C, where colours are scaled to the ratio of *NANOG* to *PDGFRA* expression. (E) Identification of variable genes for each dataset (log_2 FPKM>2 and log CV^2>0.5). (F) Intersection of variable genes common to Yan, Blakeley and Petropoulos samples.

Development • Supplementary information

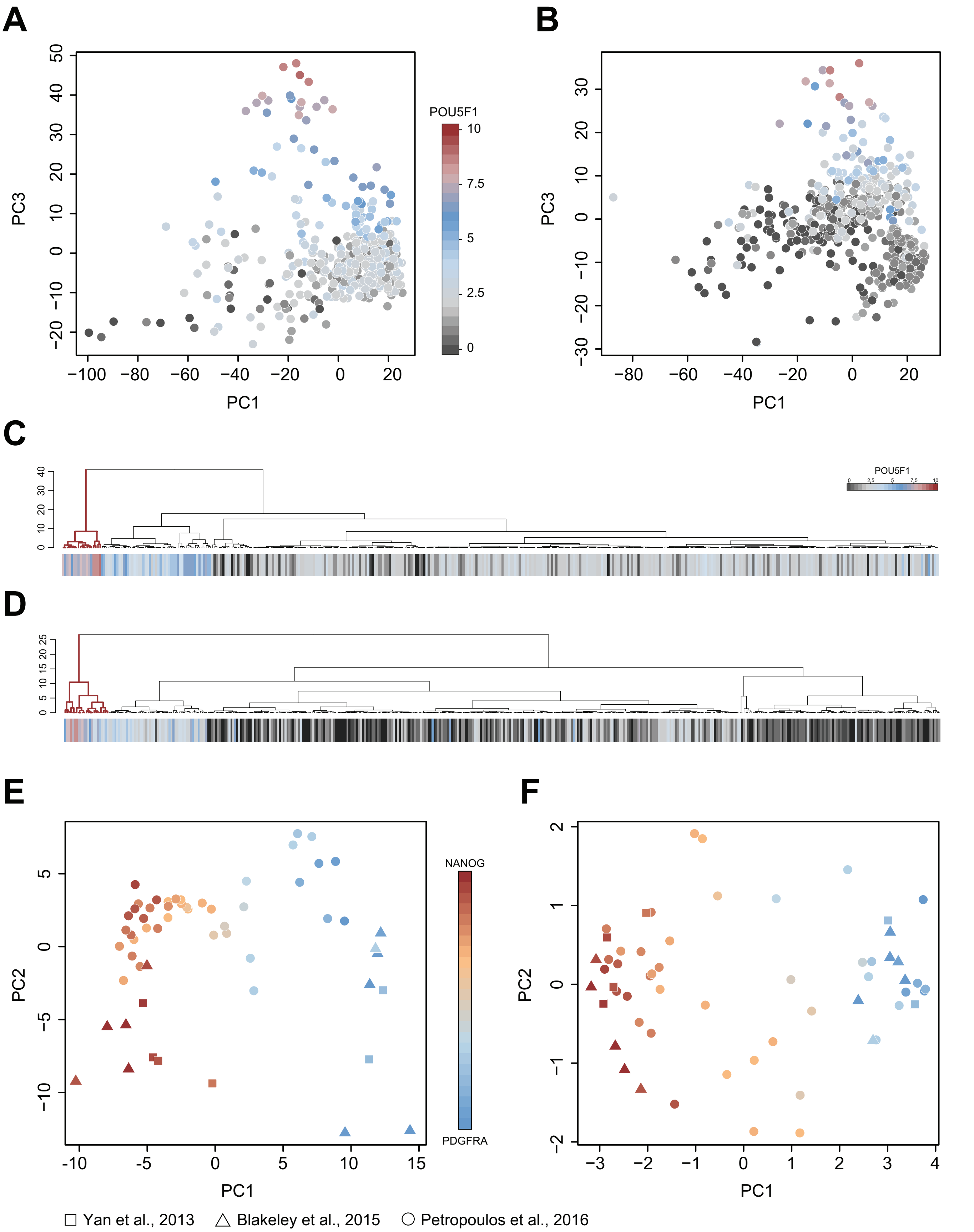

Fig. S3: (A–B) PCA of E6 (A) and E7 (B) samples from Petropoulos et al. displaying the first and third principal components. Colours represent $\log_2$ *POU5F1* expression. (C–D) Dendrograms of E6 (C) and E7 (D) Petropoulos cells based on the third principal component in B, indicating *POU5F1* expression. *POU5F1*-high clusters are highlighted in red. (E) PCA based on common variable genes (*n*=181) of Petropoulos samples with high *POU5F1* levels as selected in C and D, together with EPI and PrE cells from Yan and Blakeley datasets. (F) PCA based on high-confidence markers as described in E.

Development • Supplementary information

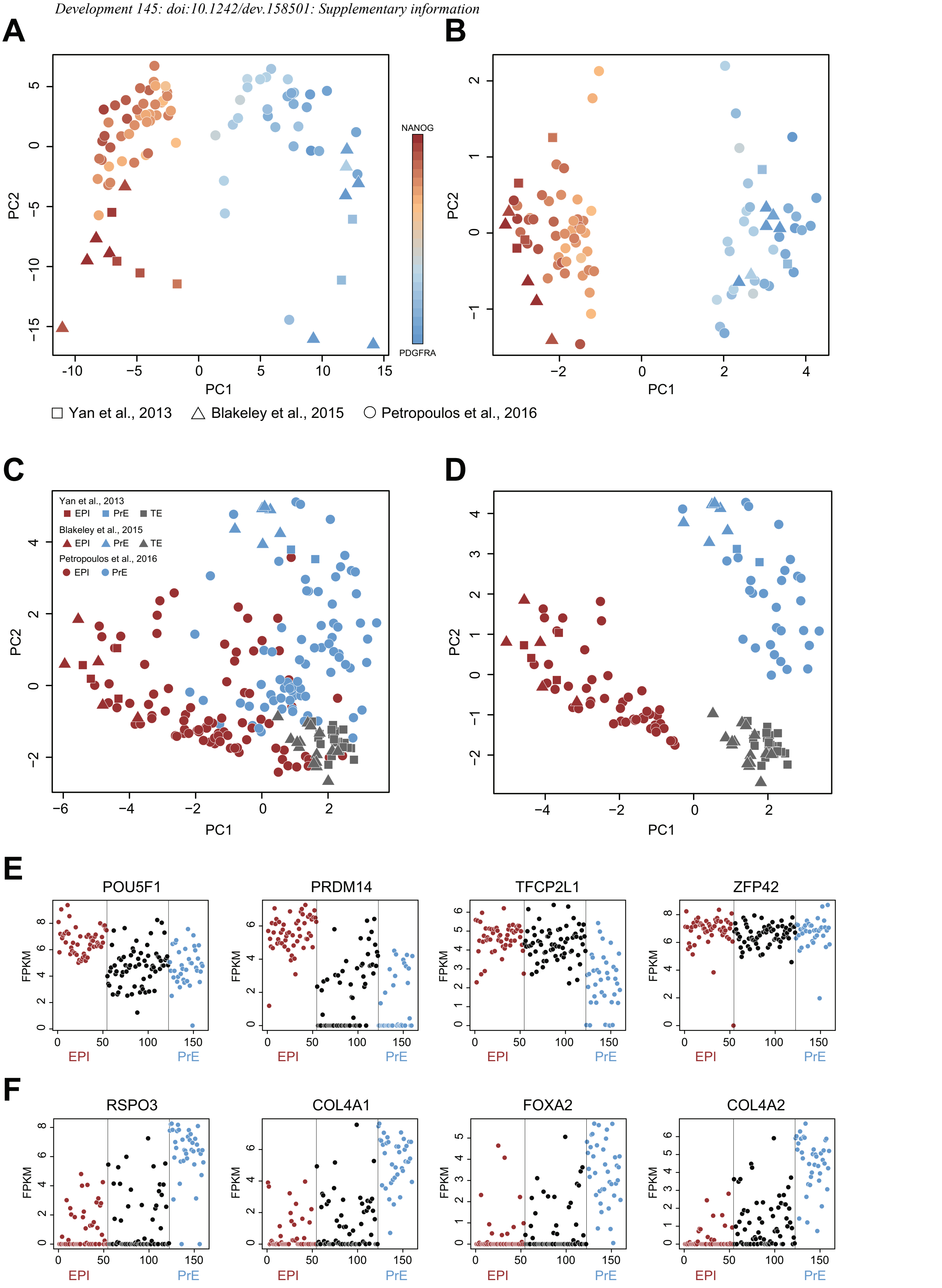

Fig. S4: (A–B) PCA for selected EPI and PrE cells based on common variable genes (A, *n*=192) or lineage markers (B). Sample colours are scaled to the ratio of NANOG to PDGFRA expression. (C) PCA based on marker genes of E6 and E7 EPI and PrE cells as classified by Petropoulos et al. (red and blue circles), together with EPI, PrE and TE from Yan and Blakeley datasets. D) PCA based on marker genes of the EPI and PrE subselection in this study, together with EPI, PrE and TE from Yan and Blakeley datasets. (E, F) Single-cell dot plots of log_2 FPKM values of the genes indicated. Cells are ordered along the *x*-axis to correspond with the dendrogram in Fig. 4A.

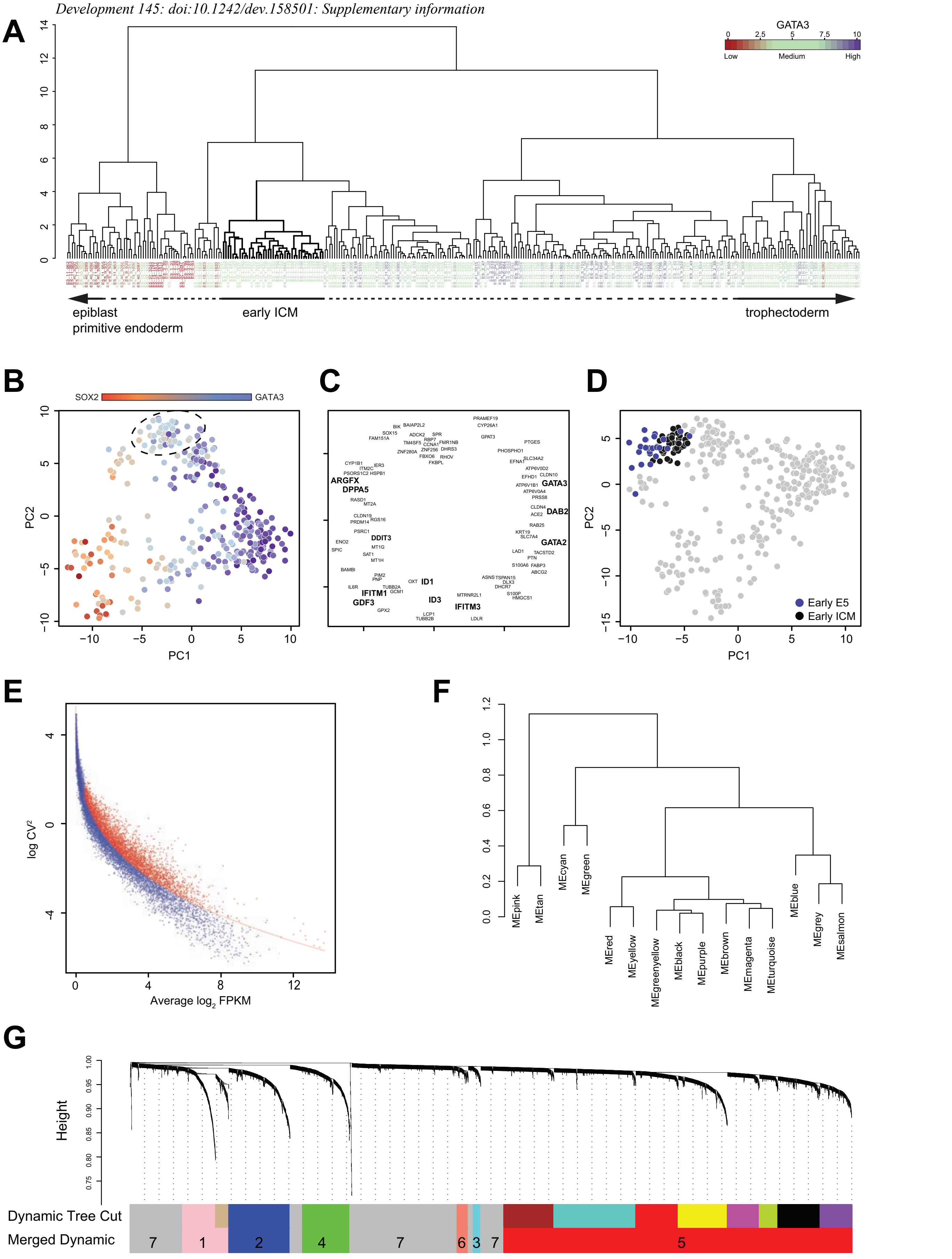

Fig. S5: (A) Dendrogram of E5 cells from the Petropoulos dataset derived from the first two principal components of PCA based on highly variable genes (*n*=203). An early ICM population is highlighted in bold. Sample colours are proportional to *GATA3* expression. (B) PCA of E5 samples from Petropoulos et al. based on variable genes (*n*=205). Samples are scaled to the ratio of *SOX2* (red) versus *GATA3* (purple) levels. (C) Spatial positions of genes contributing to the principal components plotted in B. (D) PCA of E5 samples from Petropoulos et al. based on variable genes. Early ICM cells are indicated in black, early E5 cells as annotated by Petropoulos et al. are highlighted in blue. (E) Selection of variable genes (*n*=5097) for WGCNA analysis. (F) Hierarchical clustering of the initial 14 modules identified by WGCNA. (G) Co-expression of genes and modules before and after merging.

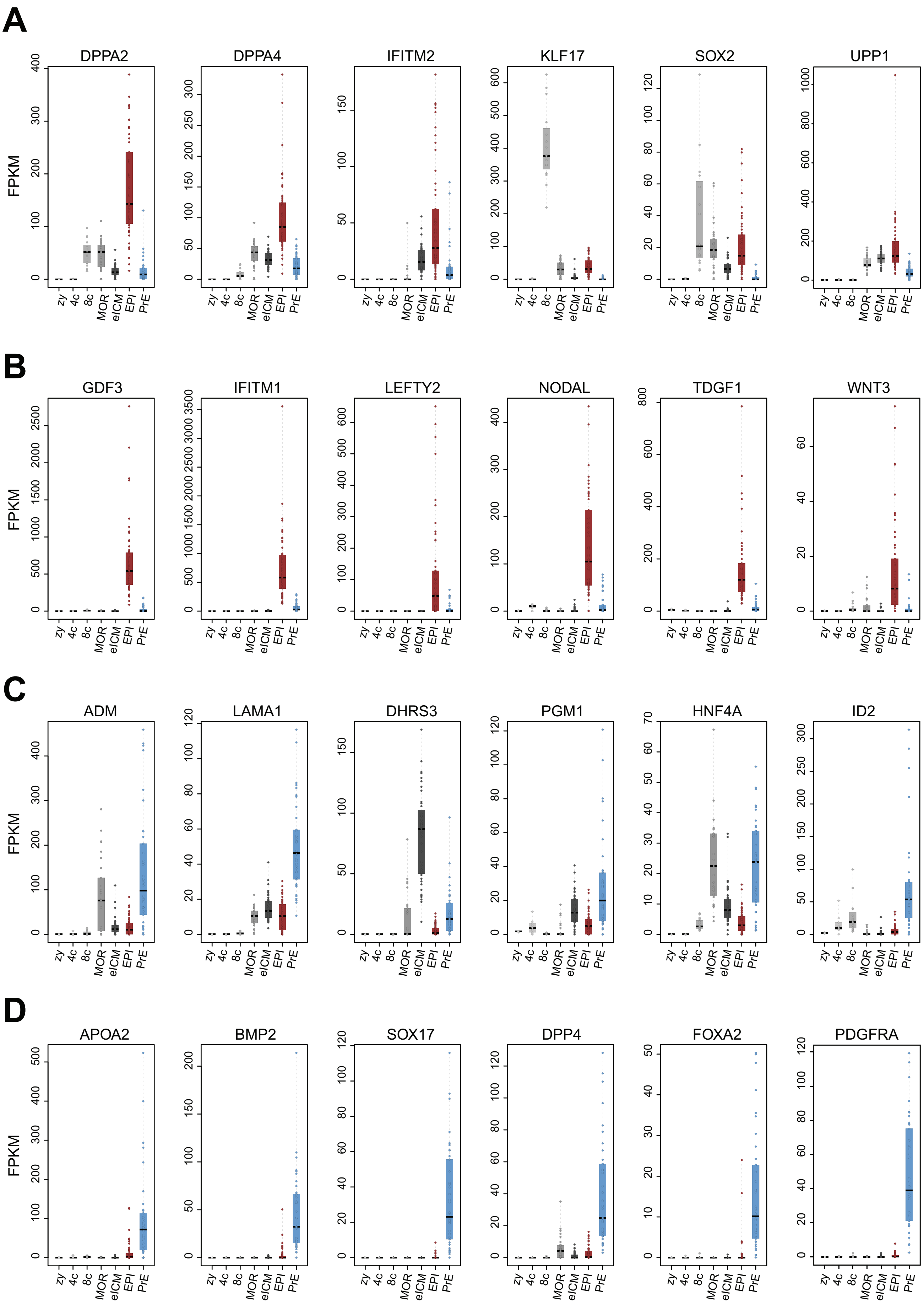

Fig. S6: (A–D) Expression in FPKM of markers for (A) early EPI, (B) late EPI, (C) early PrE and (D) late PrE.

Development • Supplementary information

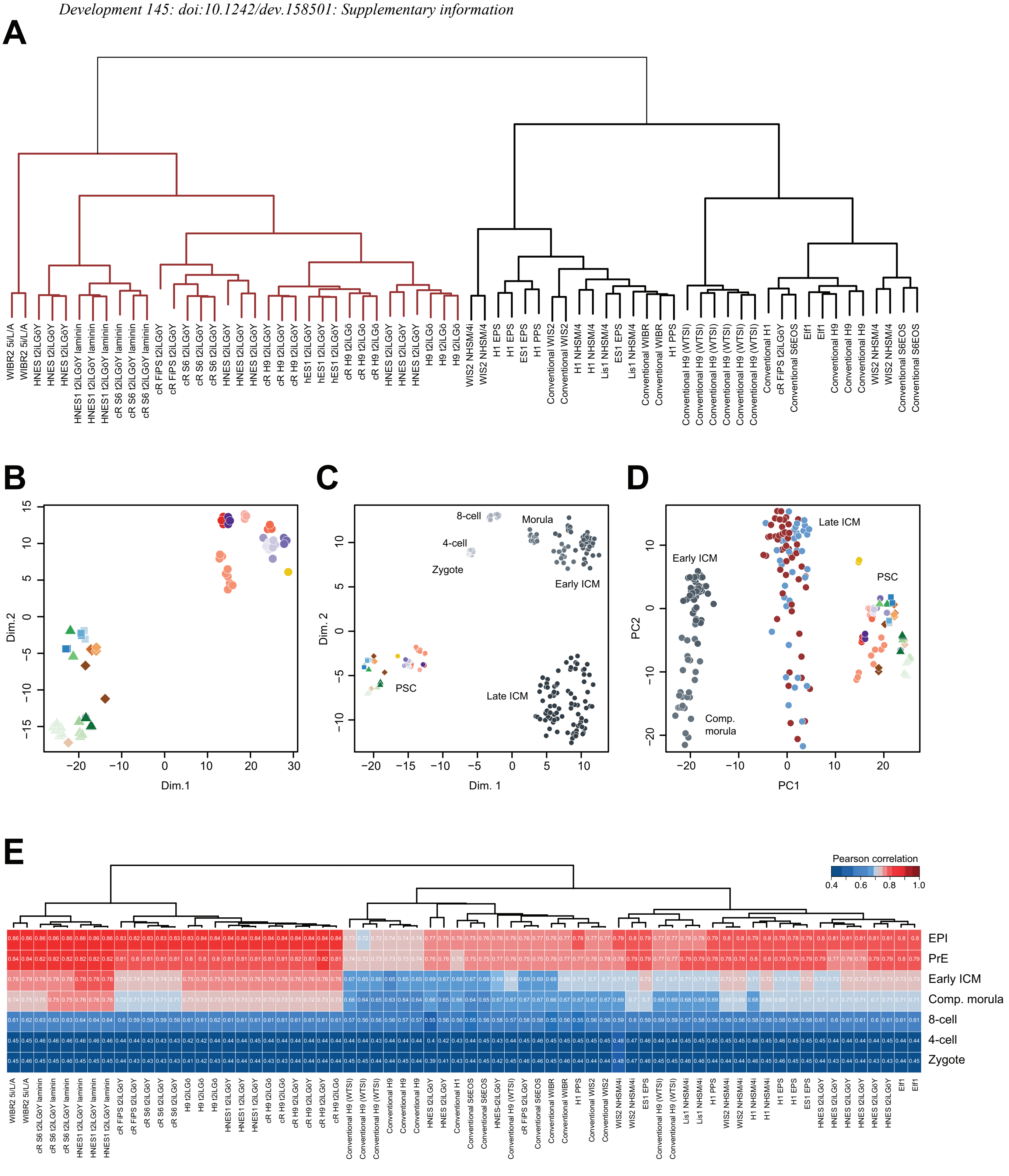

Fig. S7: (A) Hierarchical clustering of in vitro PSC samples. (B) t-SNE of PSC based on genome-wide expression. (C) t-SNE as in (B) including all preimplantation embryo stages. (D) PCA based on highly variable genes (*n*=827) of PSC cultures together with compacted morula, early ICM and late ICM samples (red=EPI, blue=PrE). (D) One-way clustering of Pearson correlation between averaged preimplantation stages and PSC cultures.

Supplementary Tables

Table S1: Differential expression analysis of Blakeley EPI, PrE and TE lineages.

Click here to Download Table S1

Table S2: Differential expression analysis of EPI and PrE cells classified in this study.

Click here to Download Table S2

Table S3: WGCNA of early ICM, EPI and PrE intramodular connectivity.

Click here to Download Table S3

Table S4: Yan, Blakeley and Petropoulos samples with originally reported and reassessed lineage assignments.

Click here to Download Table S4

Table S5: Normalised FPKM expression values for preimplantation stages.

Click here to Download Table S5

Table S6: Stage-specific gene expression modules as defined by self-organising maps.

Click here to Download Table S6

Table S7: Early and late lineage markers.

Click here to Download Table S7

Table S8: Normalised averaged FPKM expression values for preimplantation stages and PSC cultures.

Click here to Download Table S8

Table S9: Differential expression analysis of naïve versus conventional PSC.

Click here to Download Table S9

Development • Supplementary information
